# Technology‐Enabled Dementia Care Innovations: Vaccine Education Exergame for People with Dementia

**DOI:** 10.1002/alz70861_109024

**Published:** 2025-12-23

**Authors:** Winnie Sun

**Affiliations:** ^1^ Ontario Tech University, Oshawa, ON Canada

## Abstract

**Background:**

Vaccines significantly reduce the risk of various illnesses, such as Influenza, Pneumonia, and COVID‐19, all of which are among Canada’s top ten causes of death. However, vaccine hesitancy remains a concern, especially among higher‐risk populations, including older adults and persons with dementia (PWD). Enhancing current vaccine education may aid in addressing vaccine hesitancy.

**Objectives:**

This mixed‐methods pilot study aims to explore the physical, social, and educational benefits of using educational exergaming to enhance vaccine education among persons with dementia (PWD) and their caregivers.

**Methods:**

The development of the Bingo‐style vaccine education exergame was informed by a scoping review examining current digital interventions for persons with dementia (PWD) and older adults, highlighting the need for interactive and cognitively inclusive digital tools. A pilot study was then conducted with PWD and caregivers using a bingo‐style vaccine educational exergame design. Data were collected through post‐intervention surveys and semi‐structured interviews. Quantitative data were analyzed descriptively, while qualitative data was analyzed thematically.

**Results:**

Preliminary findings reveal positive attitudes towards the educational exergame, emphasizing the increase of vaccine knowledge and confidence. Furthermore, the exergame was found to be highly usable. However, challenges were noted in the complexity of vaccine education content and the length of the program.

**Conclusion:**

The study revealed that the bingo vaccine educational exergame is highly usable with physical, social, and educational benefits for persons with dementia and their caregivers. However, further research is needed to understand how varying cognitive impairments may shape user experience and design preferences.

